# Guidance on sexual, reproductive, maternal, newborn, child and adolescent health in humanitarian and fragile settings: a scoping review

**DOI:** 10.1136/bmjgh-2023-013944

**Published:** 2024-03-29

**Authors:** Mehr Gul Shah, Teesta Dey, Sophie Marie Kostelecky, Maria El Bizri, Mariana Rodo, Neha S Singh, Samira Aboubaker, Egmond Samir Evers, Per Ashorn, Etienne V Langlois

**Affiliations:** 1 The Partnership for Maternal Newborn & Child Health, Geneva, Switzerland; 2 London School of Hygiene and Tropical Medicine, London, UK; 3 Department of Global Health and Development, Faculty of Public Health and Policy, London School of Hygiene and Tropical Medicine, London, UK; 4 Independent Consultant, Bellevue, Switzerland; 5 Department of Maternal, Newborn, Child and Adolescent Health, WHO, Geneva, Switzerland; 6 Faculty of Medicine and Health Technology, Tampere University and Tampere University Hospital, Tampere, Finland

**Keywords:** Health systems, Health systems evaluation, Maternal health, Public Health, Systematic review

## Abstract

**Introduction:**

Progress related to sexual, reproductive, maternal, newborn, child and adolescent health (SRMNCAH) has stalled. COVID-19, conflict and climate change threaten to reverse decades of progress and to ensure the health and well-being of vulnerable populations in humanitarian and fragile settings (HFS) going forward, there is a need for tailored guidance for women, children and adolescents (WCA). This review seeks to map and appraise current resources on SRMNCAH in HFS.

**Methods:**

In line with the updated Joanna Briggs Institute guidance and the Preferred Reporting Items for Systematic Reviews and Meta-Analyses Extension for Scoping Reviews framework, a manual literature review was conducted of global and regional guidance published between January 2008 and May 2023 from members of the Global Health Cluster, the Global Nutrition Cluster and the Inter-Agency Working Group on Reproductive Health in Crises. A content analysis was conducted. Scores were then calculated according to the Appraisal of Guidelines for Research and Evaluation II scoring tool and subsequently categorised as high quality or low quality.

**Results:**

A total of 730 documents were identified. Of these, 141 met the selection criteria and were analysed. Available guidance for delivering SRMNCH services exists, which can inform policy and programming for the general population and WCA. Important gaps related to beneficiaries, health services and health system strengthening strategies were identified.

**Conclusion:**

The review revealed there is evidence-based guidance available to support interventions targeting WCA in HFS, including: pregnant and lactating women, women of reproductive age, adolescents, newborns, small vulnerable newborns, stillbirths, refugees and internally displaced persons and WCA with disabilities. However, gaps related to beneficiaries, health services and health system strengthening strategies must be addressed in updated guidance that is created, disseminated and monitored in a standardised way that is mindful of the need to respond rapidly in HFS.

**WHAT IS ALREADY KNOWN ON THIS TOPIC**

- Nearly one-quarter of the world’s population lives in humanitarian and fragile settings (HFS) and more than 1% has been forcibly displaced.
- Weak national health systems and high levels of insecurity have resulted in a high burden of disease and death, especially for women, children and adolescents (WCA) in HFS.
- In 2020, 43% of global under-five deaths occurred in countries classified as fragile or conflict-affected by the World Bank; the maternal mortality ratios in the world’s most fragile contexts are more than double the global average.
- To achieve the Sustainable Development Goals and Universal Health Coverage, more appropriate and cost-effective sexual, reproductive, maternal, newborn, child and adolescent health (SRMNCAH) interventions need to be designed, prioritised and delivered in HFS.
- However, with the rise of emerging shocks including COVID-19, decision-makers and practitioners lack knowledge of the existing evidence-based guidance available for use in such settings.

**WHAT THIS STUDY ADDS**

- While guidance on the SRMNCAH continuum in HFS is available, there is a scarcity of contextualised guidance for specific beneficiaries: small and vulnerable newborns, including preterm and/or low birth weight (LBW); children aged 5–9 years, adolescent boys and people with disabilities; health services: abortion, stillbirths, immunisation and adolescents health and well-being (other than sexual and reproductive health and rights (SRHR) services); and health system strengthening strategies: governance, leadership and financing.
- In the existing guidance, the use of systematic and robust methods to identify the evidence base, use of external experts, meaningful engagement of the target audience(s) and clarity around editorial independence appears more limited.
- There is also insufficient contextualisation and guidance on translating global recommendations into practical actions based on different types of HFS.

**HOW THIS STUDY MIGHT AFFECT RESEARCH, PRACTICE OR POLICY**

- Given the complex vulnerabilities and the disproportionate health burden faced by WCA in HFS, there is a critical need for clearer and more comprehensive guidance on clinical practice, public health policy and health systems arrangements to improve SRMNCAH, with a particular focus on pre-existing gaps.
- It is therefore pertinent to ensure that the guidance being developed by the global health community is tailored to different types of contexts and contextualised to overlooked beneficiary groups (small and vulnerable newborns, including preterm and/or LBW; children aged 5–9 years and adolescent boys and WCA living with disabilities), health services (abortion, stillbirths, immunisation and adolescents health and well-being (other than SRHR services) and health system strengthening strategies (governance, leadership and financing) that are not well covered in the current available guidance.
- There is also a need across the global health community, to take a more standardised approach to developing, disseminating and monitoring the uptake of guidance that is mindful of the need to respond rapidly with guidance in times of acute emergencies, but can also underscore the importance of guidance that is based on robust and consensual evidence, and both transparent and inclusive in terms of its decision-making processes.

## Introduction

As the global community crosses the halfway mark towards the 2030 Sustainable Development Goals (SDGs), any progress being made in achieving good health and well-being for women, children and adolescents (WCA) is far from universal.^1^ Gains made in reducing maternal mortality and the number of newborn and stillbirth deaths during the Millennium Development Goal (MDG) era, 2000–2015, have stalled.^2^ Little progress on the burden of preterm births—the leading cause of newborn death—has been made in two decades, with 2 million families suffering losses each year. Reduction in maternal mortality stagnated globally between 2016 and 2020. During the MDG era, however, there was an average annual reduction rate of 2.7%.^2^

The latest estimates show that, globally, a combined 4.5 million deaths—maternal deaths (0.29 million), stillbirths (1.9 million) and newborn deaths (2.3 million)—occurred in 2020.^3^ Those living in humanitarian and fragile settings (HFS) are at particular risk of falling behind. Progress is uneven, and large inequities persist. The gap between the burden of maternal, newborn, child and adolescent mortality in HFS and the rest of the world continues to grow.

This has only been exacerbated by the toxic combination of COVID-19, conflict and climate change that have threatened to reverse decades of progress made towards sexual, reproductive, maternal, newborn, child and adolescent health (SRMNCAH). It is currently estimated that a record 339 million people will require humanitarian assistance and protection in 2023.^4^ This is the highest figure in decades and a significant increase from 274 million people at the beginning of 2022 and 235 million people in 2021.^4^ At the end of 2021, more than 1% (89.3 million) of the world’s population was forcibly displaced, of whom 41% were children.^5^ Since then, that number has risen to an estimated 103 million.^6^

Delivering even the most essential health services to vulnerable and displaced populations in HFS becomes challenging in the face of high insecurity, weakened or non-existent health and social security systems, disrupted supply chains and scarce financial and human resources. In such crisis-affected contexts, WCA often bear a disproportionate burden of mortality and morbidity. Based on the most recent United Nations Inter-agency Group for Child Mortality Estimation estimates, in 2021, the rate of under-five mortality was three times higher in countries classified as fragile or conflict-affected by the World Bank than in other countries.^7^ Similarly, the maternal mortality ratio for countries classified as ‘very high alert’ to ‘high alert’ by the Fragile States Index, is over double the world average at 551 maternal deaths per 100 000 live births.^8^

Other evidence suggests that women of reproductive age living near high-intensity conflicts experience mortality three times higher than women living in peaceful settings.^9^ Up to a third of girls living in HFS report their first sexual encounter as being forced.^10^ In almost two-thirds of countries, women are more likely than men to report food insecurity, which is fuelled by conflict and climate change.^11^

Given the inequitable burden of mortality and morbidity experienced by WCA in such contexts, strengthening the delivery of, and improving access to, quality essential health services is urgent and a pre-requisite to achieving the SDGs, especially following the COVID-19 pandemic. The disruption caused by the COVID-19 pandemic is associated with an additional 113 962 deaths among women and children in 18 low/middle-income countries (LMICs).^12^

Additionally, discontinuing essential healthcare services during past epidemics resulted in more deaths than the epidemics themselves.^13^ For example, during the 2014–2015 Ebola outbreak in Sierra Leone, which killed 3500 people, an additional 3600 mothers and babies died because of the disruption to essential health services.^14^ Antenatal care coverage decreased by 22%, and there were declines in the coverage of family planning (6%), facility delivery (8%) and postnatal care (13%).^11^

Universal Health Coverage and many other SDG targets can only be achieved if urgent attention is paid to HFS worldwide and to the health needs of the WCA residing in them.

The Roadmap to Accelerate Progress for Every Newborn in Humanitarian Settings (2020–2025) noted that among the 49 countries that have experienced an acute or protracted humanitarian crisis in the past 5 years (at the time of the Roadmap’s publication), 75% were falling short of the 2030 SDG target for neonatal mortality.^15^ Similarly, 54 countries—80% located in Africa—are not on track to achieve the SDG target on reducing postneonatal child mortality to below 25 deaths per 1000 live births.^16^

To design and deliver appropriate and cost-effective SRMNCAH interventions in HFS, decision-makers and practitioners require evidence-based guidance on critical issues, including health systems arrangements, multi-sectoral coordination and emergency preparedness and response measures. To ensure vulnerable populations can be reached by essential health services, there is a need for guidance that is tailored to their needs and adapted to resource-strained settings. Existing guidance has seldom been contextualised to HFS and has been under-used when it comes to supporting evidence-based responses.^17^

This review seeks to map and appraise current guidance resources on SRMNCAH that have been contextualised to various types of HFS and are supporting policy, programmes and action. In view of the impact of emerging pandemics, including COVID-19, and subsequent emerging humanitarian and development crises, this review also seeks to include guidance tailored to pandemic preparedness and response measures as well as a broader view of multi-sectoral and health system strengthening recommendations and approaches.

## Methods

The scoping review was conducted in line with the updated Joanna Briggs Institute guidance and is reported in accordance with the Preferred Reporting Items for Systematic Reviews and Meta-Analyses Extension for Scoping Reviews framework.^18 19^

### Data sources search strategy

An initial template search matrix was developed combining search terms for the key SRMNCAH populations as exploded MeSH headings and free-text terms. Synonyms for the key search terms and Boolean operators were provided to formulate the search strategy matrix. An example of the search strategy used is presented in online supplemental annex 1.

10.1136/bmjgh-2023-013944.supp1 Supplementary data

The search comprised a manual literature review of global and regional guidance published between January 2008 and April 2023 on the English language websites of all members organisations of the Global Health Cluster (GHC), Global Nutrition Cluster (GNC) and the Inter-Agency Working Group on Reproductive Health in Crises (IAWG). 2008 was chosen as the start point primarily because this was when the WHO standardised its guidance development process.^16^ The initial literature search for this study was conducted in May 2021 (MGS and TD). To ensure the comprehensiveness and currency of our review, we conducted an additional, thorough search in May 2023 (MGS and SK). The list of organisations (n=116) can be found in online supplemental annex 2.

Additional outreach was also done to key organisations working in the humanitarian space in May 2021, including global healthcare professional associations and institutions. These organisations were identified in a desk-based mapping (n=21) and were contacted to source additional documents that might not be available online but that are used in the field.

### Definitions

Definitions of key concepts are as follows, for the purpose of this review.

#### Humanitarian and fragile settings

Humanitarian settings are defined as ones

in which an event or series of events has resulted in a critical threat to the health, safety, security, or well-being of a community or other large group of people. The coping capacity of the affected community is overwhelmed, in-country infrastructure is disrupted, and external assistance is required. This can be the result of man-made events such as armed conflicts, natural disasters, epidemics, or famine and often involves population displacement.^20^

Additionally, given the frequent overlap between HFS, the review also includes a focus on fragile settings. The Organisation for Economic Co-operation and Development characterises fragility ‘as the combination of exposure to risk and insufficient coping capacity of the state, systems and/or communities to manage, absorb or mitigate those risks. Fragility can lead to negative outcomes including violence, poverty, inequality, displacement, and environmental and political degradation’.^21^

#### Evidence-based guidance

The conceptualisation of guidance for this paper builds on the WHO’s definition of a guideline.^22^ This is an evidence-based document, containing recommendations for clinical practice, public health policy or health systems arrangements that can then inform the intended end-user on what to do to achieve the best health outcomes possible, individually or collectively, in specific situations.

### Inclusion and exclusion criteria

Global and/or regional level guidance resources in English were included if they had, (i) a clear focus on HFS; (ii) were published between January 2008 and May 2023; and (iii) included women, newborns (including stillbirths), children, adolescents and youth as a target population.

Additionally, guidance resources were included in the analysis if they addressed health system strengthening and/or public health measures in HFS and mentioned at least one SRMNCAH term in the title, table of content, executive summary or introduction. If multiple versions of the guidance resource existed, the most recent update identified in the search was included.

Guidance resources were excluded if they were only published as scientific reports, frameworks, assessment tools or standard operating procedures for the use or procurement of a commodity or as training modules and/or were national or subnational level. Additionally, guidance resources were excluded if they were formed solely on expert opinion from one organisation.

### Selection process

#### Title and abstract screening

Three reviewers (TD, MR and MGS) used the above eligibility criteria to screen titles and abstracts to identify documents in duplicate for potential eligibility.

#### Full-text screening

Two reviewers (TD and MGS) independently used the above eligibility criteria to screen the full texts for eligibility. The reviewers resolved any disagreements by discussion or with the help of a third reviewer (EVL). A calibration exercise was conducted initially (5% sample of the identified resources), prior to the full-text screening, to ensure there was alignment and a shared understanding of the inclusion criteria.^23^

### Data extraction

Data extraction was conducted by one reviewer (MGS). A second reviewer (TD) then independently assessed 10% of the sample guidance. No significant discrepancies were noted during this quality control assessment.

Data extraction focused on the date of publication; lead organisation; focus (global vs regional); type of document; type of HFS (prolonged emergency, camp settings, man-made, natural, fragile/low resource, humanitarian-development nexus, all emergencies); and beneficiary (eg, women, newborns, children, adolescents, vulnerable communities). Data were collected on the health interventions addressed (eg, sexual and reproductive health and rights (SRHR), antenatal care, intrapartum care, postnatal care, newborn care, breast feeding, immunisation, infectious diseases, non-communicable diseases, mental health, child development, injuries and trauma, nutrition). Broad interventions including SRHR and adolescent well-being were unpacked further to provide the critical detail needed for policy action. The SRHR services were unpacked in accordance with the categories within the comprehensive definition of SRHR as proposed by the Lancet-Guttmacher Commission.^24^ The categories for adolescent well-being were adapted from the adolescent well-being framework.^25^ The strategy and approach being recommended in the document was noted, for example, health system building blocks, multi-sectoral approaches, strengthening accountability, quality of care and community engagement as well as recommendations tailored to pandemic preparedness and response measures. The health systems building blocks were listed as per the WHO health systems framework.^26^

### Risk of bias assessment

Undertaking a quality assessment is not required for scoping reviews. However, because of certain circumstances, such as public health emergencies, some guidance resources are developed in rapid timeframes and through various methods (eg, on the basis of expert opinion only in the face of limited evidence). Nevertheless, there are a few foundational principles that still need to be adhered to for high-quality guidance, as described in WHO’s handbook for guideline development. For instance, ‘the recommendations should be applicable to the specified population and setting; the sources for the recommendations must be indicated; to the extent possible, efforts must be made to minimize the risk of bias; and the development process should be explicit and transparent’.^21^

An assessment of guidance quality was thus undertaken, using the Appraisal of Guidelines for Research and Evaluation (AGREE) II instrument.^27^ This tool is well-established and was widely used in the literature to assess guidance quality. Two reviewers (MGS and TD) independently reviewed all documents. The tool has the following six domains: scope and purpose (n=3), stakeholder involvement (n=3), rigour of development (n=8), clarity of presentation (n=3), applicability (n=4) and editorial independence (n=2). In cases of non-consensus, advice was sought from a third reviewer (EVL).

### Data synthesis and analysis

A narrative synthesis was undertaken where each selected guidance resource was reviewed and data under each of the headings was extracted. Data were analysed using content analysis and grouped in terms of frequency. The frequency of responses for each of the background and data categories for all included resources were recorded, synthesised and presented through data visuals. For the outcomes, guidance resources would cover multiple themes and non-mutual categorisation was used for these groupings.

Following the agreement of AGREE II scoring, six domain-specific scores were calculated and expressed as a percentage of the theoretical maximum for that domain. Based on the scoring, guidance articles were then categorised as high or low quality. Categorisation was based on established approaches within the existing literature, whereby if four or more domains scored >50%, the guidance article was deemed of high quality.^25^

### Patient and public involvement

Patients were not involved in the development of this scoping review and paper.

## Results

**Figure 1:**
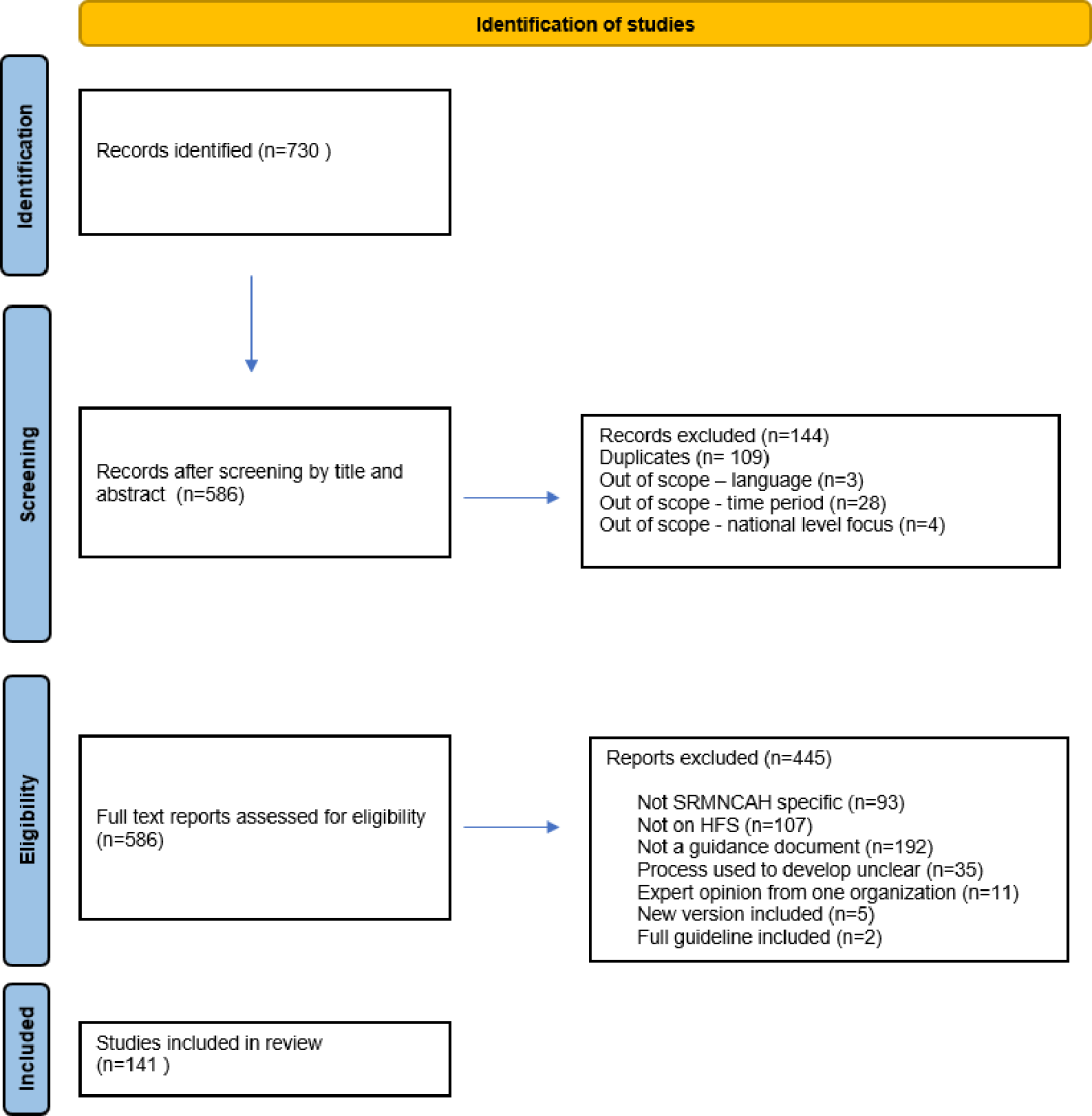
Preferred Reporting Items for Systematic Reviews and Meta-Analyses Extension for Scoping Reviews flowchart diagram. HFS, humanitarian and fragile settings; SRMNCAH, sexual, reproductive, maternal, newborn, child and adolescent health.

A total of 730 documents were identified using the search strategy (figure 1). Of these, 141 met the selection criteria and formulated the body of guidance on SRMNCAH in HFS that was then analysed (see online supplemental annex 3 for the list of included documents).

### Description of included resources

**Figure 2:**
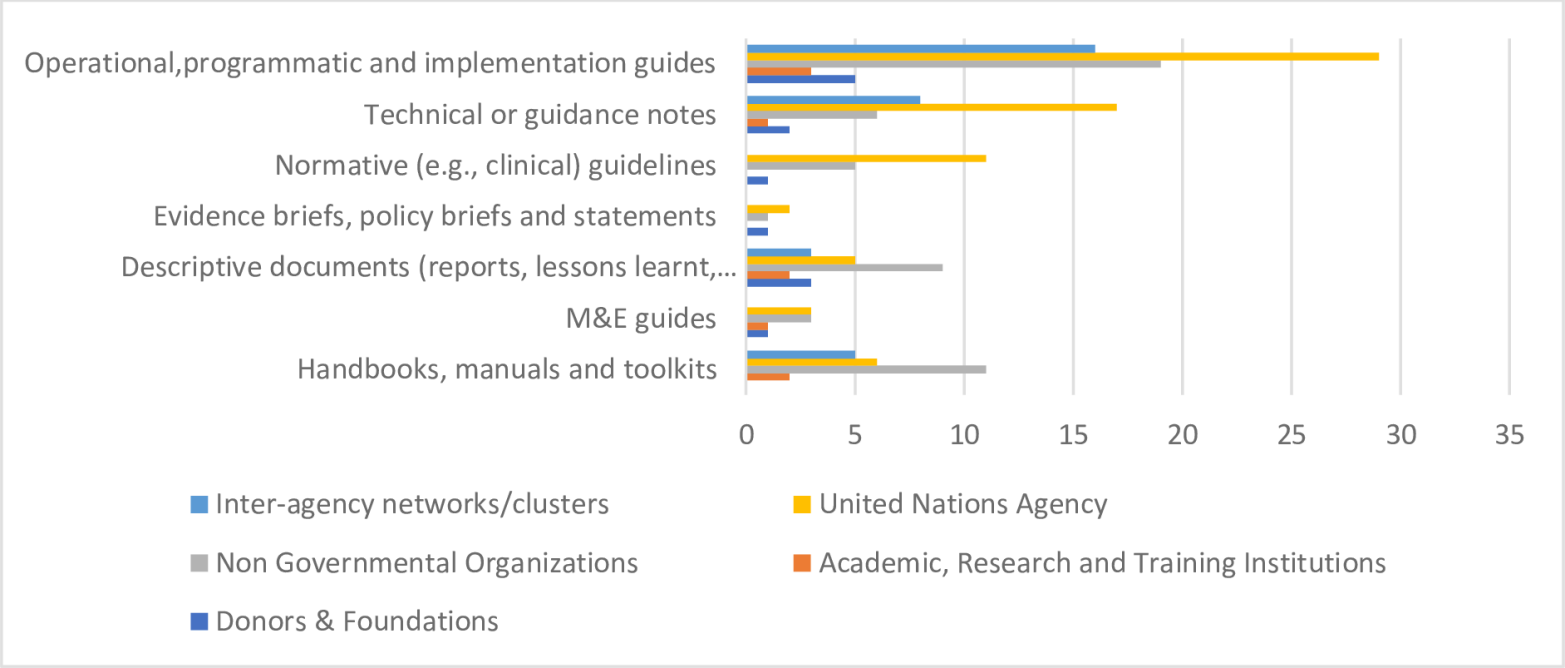
Spread of guidance resources across constituencies/stakeholder groups.

The guidance was categorised into different types of resources, as listed in figure 2. The majority were classified based on the title description, abstract and acknowledgement or introduction of the document. Most resources were categorised as operational, programmatic or implementation guides (n=59, 42%). Over 40% of resources self-described as technical/guidance notes (n=22, 16%), handbooks, manuals and toolkits (n=21, 15%), clinical guidelines (n=15, 11%) or descriptive reports (n=14, 10%). Most resources were global (n=136, 96%). Over a quarter had been created or updated since 2020 (n=55, 39%). Guidance resources were developed (at times jointly) by four key constituencies: United Nations (UN) agencies; academic, research and training institutes; non-governmental organisations (NGOs); or donors and foundations (figure 2). Over half of resources were created by a UN agency (n=73, 52%), followed by (international) NGOs (n=56, 40%). Additionally, almost a quarter (n=34, 24%) were created by inter-agency working groups or mechanisms such as the GNC, Inter-agency Standing Committee or the IAWG.

### Univariate outcomes

Most resources (n=46, 33%) involved an overarching focus on the general population as beneficiaries, with either a sub-focus on SRMNCAH services or WCA. 27 resources had guidance pertaining to pregnant and lactating women (19%) and 10 were focused on women of reproductive age (7%). Almost one in six resources had guidance pertaining to adolescents (n=25, 17%), yet only three (2%) were focused on adolescent boys. 37 resources (26%) had guidance on newborns; however, only four (3%) were focused on small vulnerable newborns.^28^ Only two resources (1%) included a sub-focus on stillbirths. Refugees and internally displaced persons (IDPs) were included as beneficiaries in close to a fifth of resources: 29 (20%) and 20 (14%) resources, respectively. 17 (12%) resources included some form of guidance for WCA living with disabilities.

Within the 25 resources for adolescents, most resources had guidance on adolescent physical health and capacities (n=17) and 11 resources included a focus on adolescent mental health. Five resources had guidance on optimal nutrition status and diets.

Only 10 resources (7%) included a focus on lesbian, gay, bisexual, transgender, queer or questioning, or another diverse gender identity (LGBTQ+) and seven resources (6%) included Indigenous communities and/or religious/ethnic minorities as beneficiaries.

Over half of the available guidance had a focus on SRHR services (n=73, 51%) followed by nutrition, diet and feeding (n=37, 26%) and infectious diseases (n=30, 21%). Less than 10 guidance resources covered antenatal care (n=8, 5%), injuries and trauma (n=6, 4%), immunisations (n=3, 2%) and non-communicable diseases (n=2, 1%).

**Figure 3:**
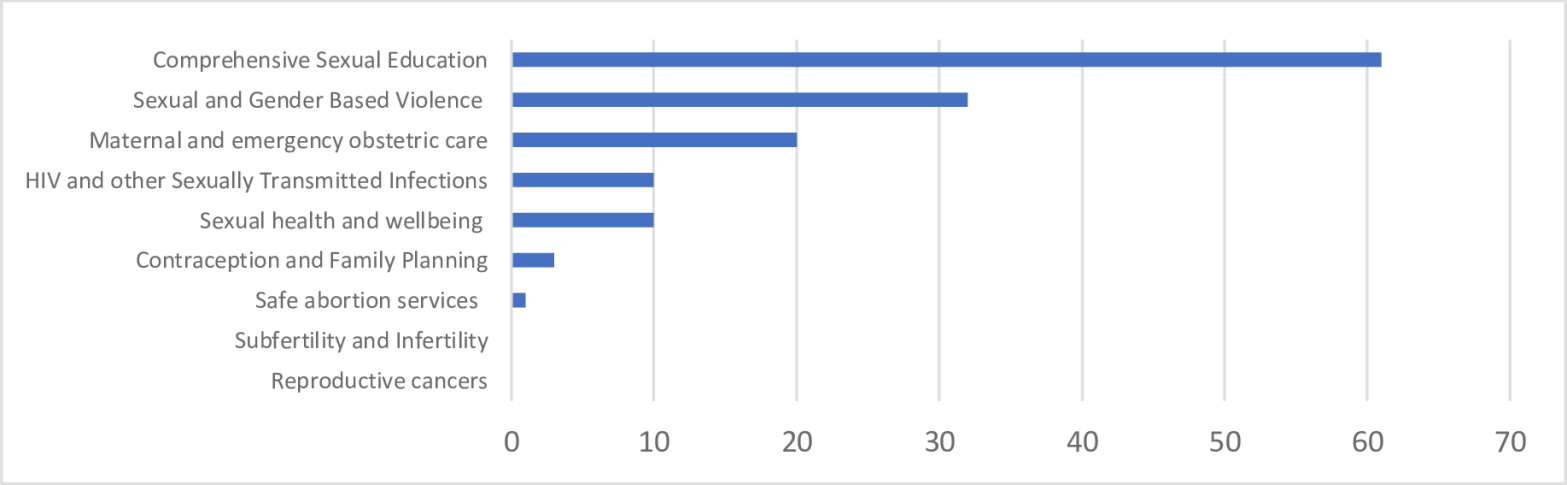
Spread of resources by type of sexual and reproductive health and rights service.

The 73 resources that had focussed on SRHR services were further analysed in accordance with the categories within the comprehensive definition of SRHR as proposed by the Lancet-Guttmacher Commission (figure 3).^22^ Most of the SRHR resources (n=61) provided guidance to some extent on comprehensive sexuality education. 31 provided guidance on sexual and gender-based violence while 20 resources provided guidance on antenatal, childbirth and postnatal care, including emergency obstetric and newborn care. Only one resource provided guidance on safe abortion care and no resources looked at reproductive cancers or sub-fertility and infertility.

With regard to the health building blocks, the majority of the resources (n=118, 84%) provided guidance around service delivery, health information systems (n=37, 26%) and the health workforce (n=21, 15%). Less than 10% of resources looked at health systems financing (9%), medical products, vaccines and technology (n=11, 8%) and leadership and governance (n=9, 6%).

When looking at multi-sectoral approaches, a third of guidance focused on nutrition (n=47, 33%), 31 (22%) and 28 resources (20%) included a focus on protection and education respectively, 20 (15%) on WASH and 18 (12%) resources looked at the intersection of health and child protection.

With reference to guidance developed for different types of humanitarian settings and emergencies (eg, conflict, natural disasters, camp settings, fragile settings and emergencies), 94 (67%) resources provided guidance for all kinds of emergencies without further contextualisation. 17 (12%) were contextualised to camp settings and seven (5%) resources looked, in some part, at the transition from humanitarian to development contexts. Only three (2%) resources included guidance on natural disasters. 13 (9%) guidance resources were developed for all types of settings (development and humanitarian), but included certain recommendations for humanitarian emergencies and contexts. 13 (9%) resources were tailored to the COVID-19 pandemic and 28 (20%) provided some guidance on emergency preparedness and response strategies and measures.

### Bivariate outcomes

**Figure 4:**
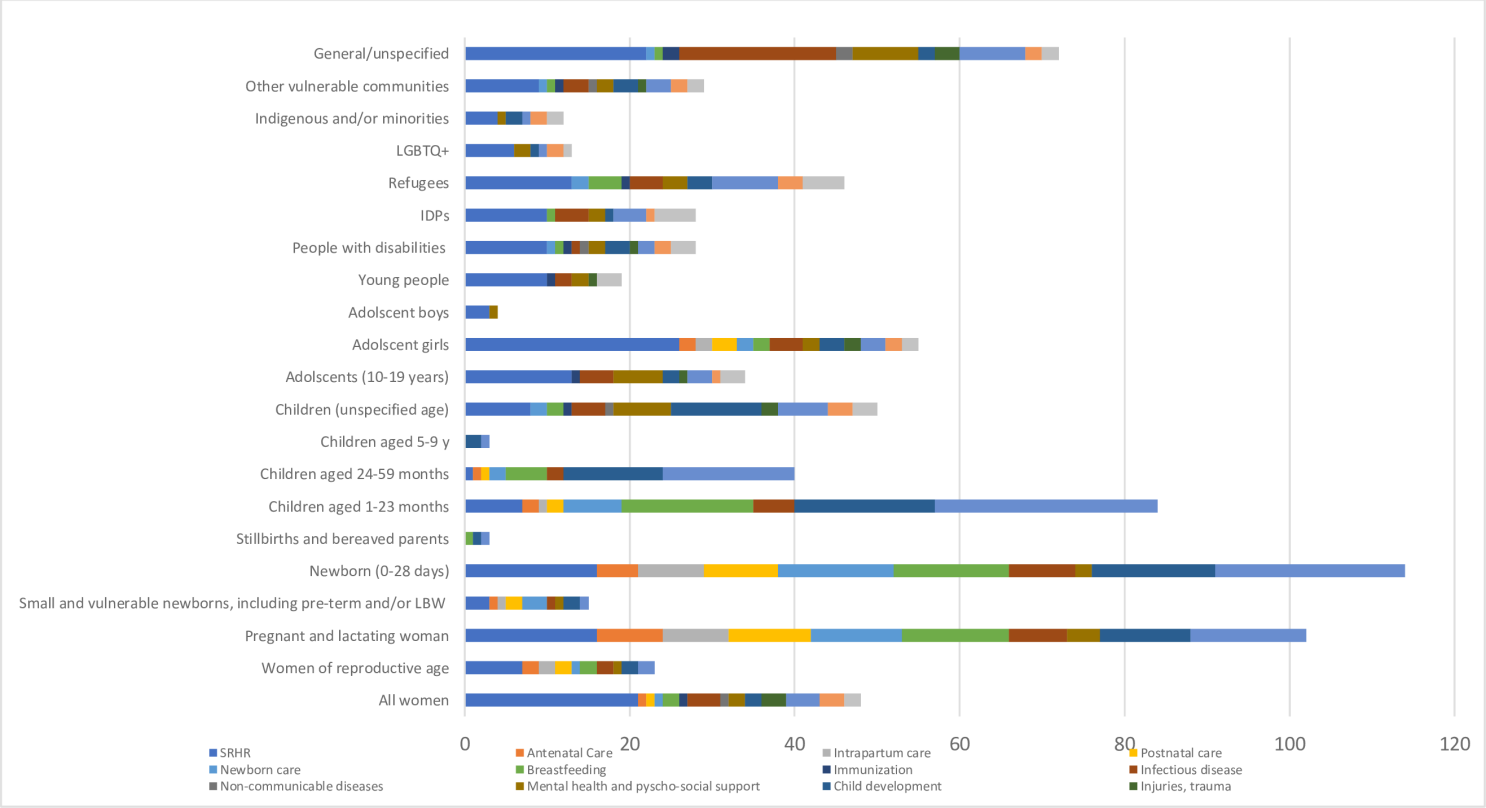
Distribution of guidance documents by beneficiary group and intervention type. The horizontal axis of figure 4 represents the number of resources or guidance documents that pertain to each specified population beneficiary. IDPs, internally displaced persons; LQBTQ+, lesbian, gay, bisexual, transgender, queer or questioning, or another diverse gender identity.

Individual interventions were analysed by population beneficiaries (figure 4). For vulnerable communities, especially LGBTQ+ and Indigenous minorities, there was a paucity of guidance on essential SRMNCAH services, with almost all guidance for these beneficiary groups focusing on SRHR. Similarly, for adolescents, the majority of guidance was related to SRHR (n=13) followed by mental health and psychosocial support (n=6). For pregnant and lactating women, guidance focused on SRHR (n=16) and maternal health services. Antenatal care and intrapartum care were a sub-focus of 8 resources, and 10 guidance documents included some level of guidance on postnatal care. 14 resources focused on newborn care, and nutrition was the most popular intervention looked at for children aged 1–23 months (n=27), followed by nutrition (including breast feeding) for newborns (n=23). There was a lack of guidance on mental health and psychosocial support for pregnant and new mothers, and only one resource looked at injuries and trauma (focusing on female genital mutilation specifically) for children and adolescents.

**Figure 5:**
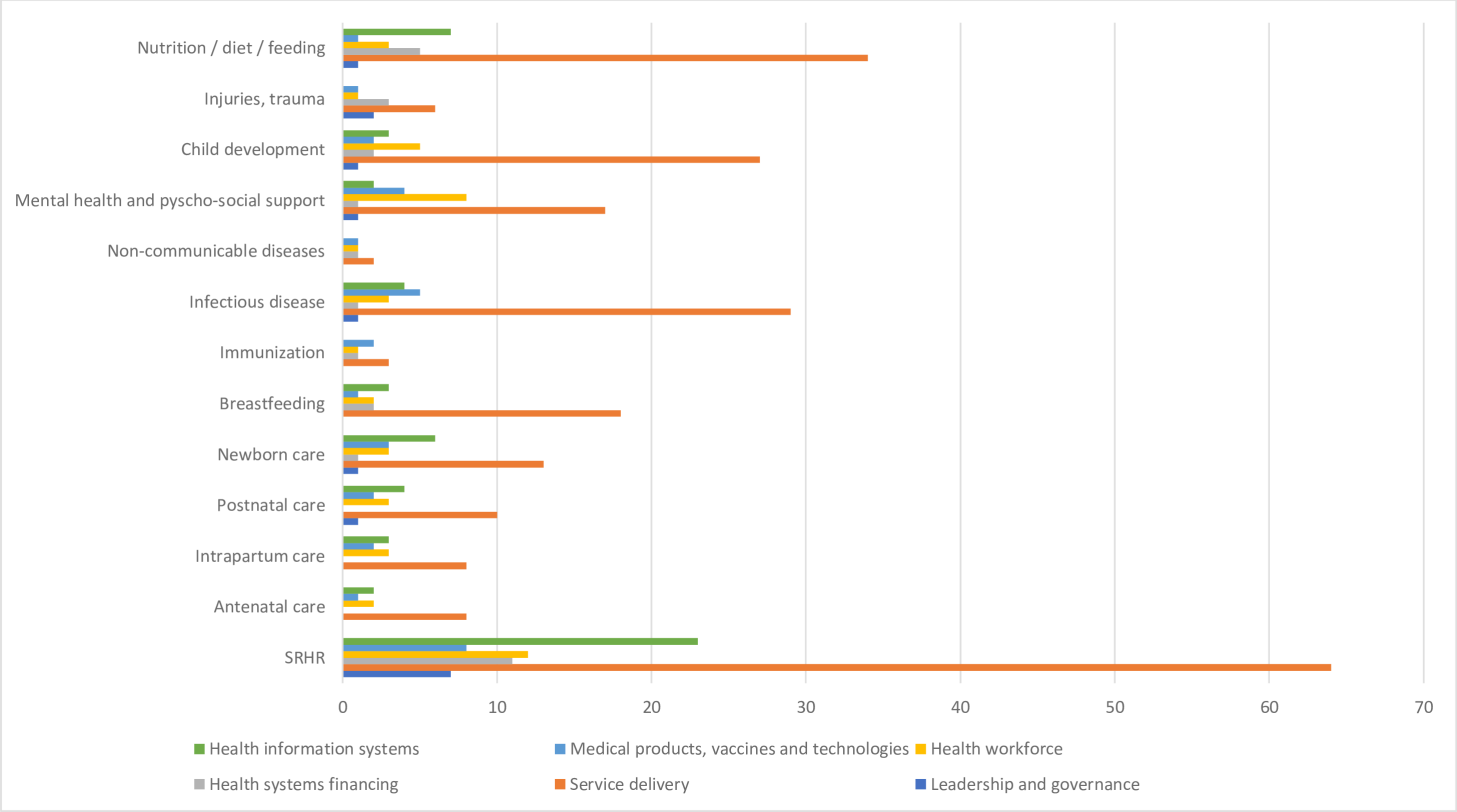
Distribution of guidance documents by intervention type and health system strengthening strategies. In figure 5, the horizontal axis quantifies the guidance documents that correspond to each health system building block as they relate to different service types.

Individual health system strengthening strategies (ie, health system building blocks) were analysed by intervention or service type (figure 5). The majority of resources for SRHR provided guidance on service delivery (n=64), health information systems (n=23) and the health workforce (n=12). Within the resources focusing on service delivery, there were 34 on nutrition and 29 on infectious diseases. Within the resources for medical products, vaccines and technologies five resources focused on infectious diseases. No resources looked at health systems financing for antenatal care, intrapartum care or postnatal care specifically, however 11 resources looked, at least partly, at financing for SRHR. There was a scarcity of guidance around strengthening governance and leadership.

### Quality assessment

A total of 46 documents met the predefined criteria to be categorised as a high-quality guidance resource. A subgroup analysis was undertaken of these documents. Trends noted in the subgroup analysis largely followed that of the broader data set for beneficiary, type of intervention, health system building blocks and multi-sectoral integration. The only contrasting feature identified was the document type where 72% (n=33) of documents within the subgroup were operational guidance.

Within the 46 high-quality guidance resources, all resources scored >50% for domain 1 ‘scope and purpose’ and domain 4 ‘clarity of presentation’. Domain 5 ‘applicability’ also scored well with 44 (96%) of the 46 resources scoring above 50%. However, 38 (82%) of the resources scored <50% for domain 3 ‘rigour of development’ and 25 (54%) resources scored <50% for domain 6 ‘editorial independence’.

## Discussion

The review of SRMNCAH guidance revealed that there are numerous evidence-based guidance resources available to support interventions targeting WCA in HFS and that they cover a wide range of beneficiaries including: pregnant and lactating women, women of reproductive age, adolescents, newborns, small vulnerable newborns, stillbirths, refugees and IDPs and WCA with disabilities.

The majority of guidance identified for SRHR focused on service delivery, health information and the health workforce, in addition to nutrition, infectious diseases, medical products and financing, to varying degrees. Over 25% of the resources had been created or updated since 2020, signifying the international community’s recognition of the urgency to address the needs of WCA living in HFS. Although this shows that an abundance of guidance already exists to guide action, most resources focused on the nutrition, followed by protection (including child protection and GBV) and education. This review also identified important gaps among guidance documents in terms of beneficiaries, health services and health system strengthening strategies. Addressing these gaps will be crucial in promoting the health and well-being of WCA in HFS.

For health services, there are critical gaps in guidance around abortion, stillbirths, adolescents’ health and well-being (beyond SRHR services). While the authors of this paper are cognisant that a single good quality guidance document could potentially be sufficient in covering a guidance gap, none of the resources that met the quality assessment criteria looked at abortion or stillbirths. In terms of beneficiaries, there is a lack of contextualised guidance for small vulnerable newborns, including preterm and low birth weight (LBW); for children aged 5–9 years; adolescent boys; and WCA living with disabilities. These findings are consistent with a review that some of the coauthors of this paper undertook to determine the amount, scope and quality of global guidance for the promotion of WCAH in conflicts from 2008 to 2018. The findings from this paper confirm that not much has changed in the 5 years since.

With reference to health strategies, while a majority of the resources focus on providing guidance around service delivery, key gaps were noted around health financing and governance and leadership in HFS.

One notable challenge in HFS, particularly relevant to health system strengthening strategies, is the predominance of short-term funding cycles. These cycles often lead to fragmented service delivery and undermine the sustainability of health interventions in humanitarian context.^29 30^ This underscores the need for guidance that addresses the continuity of health services amidst fluctuating funding landscapes. This is especially critical since climate-induced disasters, conflict and infectious diseases are driving humanitarian needs to unprecedented levels, putting additional stress on many chronically fragile LMICs.

In such contexts, effective governance and leadership are also critical to ensuring the timely delivery of quality health services, especially to the most vulnerable WCA. Research conducted by the BRANCH Consortium has documented that countries, specifically those experiencing conflict, face many challenges related to governance and leadership. Power imbalances between national and international humanitarian actors, and the lack of evidence-based decision-making, has been shown to have had an impact on the provision of health services in certain contexts.^31 32^

The intricacies of health systems within humanitarian contexts are compounded by the addition of humanitarian actors to pre-existing health infrastructures. This often results in a fragmented health system, where services may be duplicated, and resources spread thin, undermining the overall efficacy of health interventions. The interplay between myriad actors—ranging from international NGOs to local health authorities—can lead to conflicting priorities and coordination challenges, which are exacerbated in crises where health needs are acute and rapidly changing.^29 33^ The resulting fragmentation calls for a concerted effort towards integration and the strengthening of health systems governance, which is often overlooked in guidance documentation. Such efforts should aim to enhance the resilience of health systems by promoting cohesive strategies that align with both local needs and the capacities of the existing health infrastructure.^30 34^ The authors of this paper are cognisant that the Health Cluster plays a pivotal role in this regard, striving to coordinate efforts and mitigate service fragmentation. However, there is a need for more nuanced guidance that specifically addresses the challenges of governance and leadership in these settings, considering the unique dynamics of humanitarian crises.

Ensuring effective governance and leadership is a complex issue, especially in HFS where contexts can differ. As has been suggested in recent literature,^35 36^ ensuring that strategic governance conditions are maintained in such settings would enable various actors on the ground to work together more cohesively while being flexible enough to adapt to a rapidly evolving context. Global agencies and NGOs leading humanitarian responses could therefore consider collaborating with academics and community representatives to generate guidance for governance, leadership and health financing.

This review has also shown that where guidance exists, the scope and purpose of the documents are generally described well, but the use of systematic and robust methods to identify the evidence base, use of external experts and clarity on the process for developing recommendations are often unclear. This may indicate lack of robust and contextualised evidence, but equally may indicate a lack of resources available to develop guidance through an evidence-based and robust process. It was generally observed that documents led by the WHO articulated the evidence-based process used to develop the guidance, in line with the WHO’s handbook for guideline development. Stakeholder interviews conducted in a previous review^16^ found that civil society representatives felt the need for ‘balance between practical experience and academics’ and considered field experience to be a critical component in developing guidance tailored to humanitarian situations.

A key finding in the quality assessment also pointed to a lack of meaningful engagement of the target audience within the development of the guidance. Increasingly, the value of community engagement is being recognised. Community engagement ensures that guidance is meaningful and useful to the communities it is designed to benefit.^37^ This approach is well established within the WHO Guideline Development Manual but, as the review highlights, is not consistently adhered to in the development of guidance. This gap is particularly noteworthy in humanitarian settings where local actors offer unique insights due to their better understanding of the local context, closeness and accessibility to communities, established trust, and long-term reliability and accountability.^38^ Additionally, in many humanitarian settings, a significant portion of health services is delivered by local organisations that may not be directly involved in guideline development. This can lead to a discrepancy between the guidelines and actual service delivery, especially in areas with severe access or security constraints. Engaging these actors in guideline development can ensure more contextually relevant, culturally sensitive and community-aligned interventions.

The influence of authorship on the content of guidance documents cannot be understated. Given that a significant proportion of guidelines originate from UN agencies that are dependent on donor funding to undertake crucial work, it is conceivable that the content and focus of the guidelines could potentially be influenced by their operational priorities. This influence is particularly evident in our quality assessment, where 54% of resources scored below 50% for editorial independence, suggesting a possible impact of donors on content. Indeed, recent literature highlights how humanitarian responses, including guidelines, often align with donor priorities and may not fully encompass the complexities of health systems in conflict settings.^39^ Furthermore, studies have demonstrated that donors’ political agendas can significantly influence intervention delivery and access in specific geographic locations, with restrictions on the types of funded activities affecting the spectrum of services available.^40^ Our review, therefore, emphasises the necessity for diverse authorship in guideline development, including local health authorities and community representatives, to counterbalance potential biases and ensure guidelines are relevant and applicable to the unique challenges of health systems in humanitarian contexts. This approach is crucial for addressing the gaps in guidance for vulnerable populations and interventions, as identified in our outcomes section.

Increasingly complex humanitarian situations are a reality of current times. For instance, 14 of the 25 countries that are considered to be the most vulnerable to climate change are also experiencing conflict.^41^ The intersection of climate risks and conflict further exacerbates health disparities, limits WCAs’ access to essential health services and weakens the capacity of governments, institutions and societies to provide support. There is therefore a need for a standardised approach to developing guidance for HFS that can be adapted by humanitarian and development actors. A more standardised approach would help to ensure that any guidance and recommendations developed are balanced in their approach to considering expert opinion, especially that of local actors, and are evidence-based to ensure the global health community does not inadvertently violate the ‘do no harm’ principle.

Analysis from a previous review focused on global guidance around SRMNCAH in conflict settings has also shown that organisations do not typically follow standard procedures for guideline dissemination, updates or uptake monitoring.^16^ This perceived lack of prioritisation on dissemination and monitoring not only limits the use of guidance but makes it difficult to document the impact of said guidance in crisis contexts.^16^ The global community should routinely monitor the uptake and use of existing guidance, and document and amplify good practices in the adaptation and implementation of guidance for decision-making in HFS. This could potentially also inform better practices for optimising care, enhancing coordination and fostering better SRMNCAH outcomes in HFS.

The BRANCH Consortium recommends convening a technical advisory group to guide the development of an evidence-based, decision-making framework for the selection of interventions in HFS, to develop a common set of indicators for humanitarian health action and to create implementation research priorities to help fill key gaps in operational and implementation guidance.^42^ The findings from this review underscore this recommendation. They also highlight the need for guidance on the SRMNCAH continuum to be prioritised, developed through a robust and evidence-based process and contextualised to different types of humanitarian crises.

### Limitations

There were a few limitations to this work.

First, the literature search was limited to the English language and primarily focused on resources from key global organisations in the humanitarian sector, such as members of the global coordination mechanisms like the GHC, the GNC and IAWG. These organisations are considered pivotal actors within this space. This approach was taken to ensure the feasibility and manageability of our review, concentrating on global and regional guidance. It is important to note that while national and local organisations play a crucial role at the forefront of providing humanitarian assistance, their specific guidance documents fall outside the scope of this review.

As mentioned in the discussion, understanding the effectiveness and impact of the global guidance documents available, merits an analysis that focuses more deeply on what is being used in the field and how that compares to the array of global level guidance that is available widely.

In addition, the methodology followed that of pre-existing methods used to review humanitarian guidance.^16^

It was also often difficult to pinpoint a singular focus of the guidance as many of the documents covered multiple themes. As such, a non-mutually exclusive count was undertaken to ensure all nuances were captured.

Furthermore, defining the focus of guidance was prone to misinterpretation. To mitigate this, two independent researchers were used to check data abstraction for agreement.

Finally, the appraisal of quality of guidance resources was undertaken using the AGREE II tool. This tool is primarily designed to assess the quality of clinical practice guidelines. This review incorporated a range of guideline resources beyond clinical guidelines. As such, the framework used may not have been the best fit. Unfortunately, there are no frameworks developed holistically for guidance resources and so a pragmatic approach was taken to use the AGREE II tool. This limitation must be acknowledged when reviewing the guidance resources deemed ‘high quality’.

## Conclusion

Given the complex vulnerabilities and the disproportionate burden WCA face in HFS, providing guidance on clinical practice, public health policy or health systems arrangements to improve SRMNCAH, especially where there are gaps, as noted in this review, is the need of the time. However, it is also critical to ensure that the guidance being developed by the global health community, is tailored to different types of contexts and contextualised to beneficiary groups (small and vulnerable newborns, including preterm and/or LBW; children aged 5–9 years and adolescent boys and WCA living with disabilities), health services (abortion, stillbirths, immunisation and adolescents health and well-being (other than SRHR services) and health system strengthening strategies (governance, leadership and financing) that are not well covered in the current available guidance. Additionally, meaningful involvement of local actors in the development of these guidelines is vital. Their unique perspectives, stemming from an intimate understanding of and trust within their communities, are crucial for creating effective and relevant guidance in humanitarian settings.^38^

There is also a need across the global health community, to adapt a standardised approach to developing, disseminating and monitoring uptake of guidance that is mindful of the need to respond rapidly with guidance in times of acute emergencies, but can also underscore the importance of guidance that is based on robust and consensual evidence, and transparent and inclusive decision-making processes.

## Data Availability

Data are available in a public, open access repository.
