# Supplementary material for: Guidance on sexual, reproductive, maternal, newborn, child and adolescent health in humanitarian and fragile settings: a scoping review
Source: BMJ Glob Health. 2024 Mar 29;9(3):e013944. doi: 10.1136/bmjgh-2023-013944 (PMC10982774; doi:10.1136/bmjgh-2023-013944)
Supplement: Supplementary data [file bmjgh-2023-013944supp001.pdf]

Appendix 1-3: Guidance on sexual, reproductive, maternal, newborn, child and adolescent health in humanitarian and fragile settings: A scoping review

Annex 1: Search terms

1. Sexual
2. Reproduction
3. Reproducing
4. Reproductive
5. Contraception
6. Family planning
7. Sex
8. Sexual violence
9. Rape
10. Gender
11. Gender –based violence
12. Post-exposure prophylaxis
13. Abortion
14. Maternal
15. Mother*
16. Pregnancy
17. Delivery
18. Birth*
19. - ANC
20. - Antenatal
21. - Ante-natal
22. - Ante natal
23. - Neonatal
24. - Neonate*
25. - Newborn*
26. - Baby*
27. - PNC
28. - Postnatal
29. Post-natal
30. Stillbirth*
31. Child
32. Children
33. Infant *
34. Young Child
35. Toddler*
36. Adolescent*
37. Adolescence
38. Teenage
39. Teenager*
40. Teen*
41. Youth*
42. Young people
43. Young person
44. Nutrition
45. Feeding
46. Food
47. Undernutrition
48. Malnutrition

49. Malnourishment
50. Severe acute malnutrition
51. SAM
52. Moderate acute malnutrition
53. MAM
54. Breastfeeding
55. Lactation
56. Humanitarian
57. Emergency
58. Fragile settings
59. Low resource

Search Strategy:

1 or 2 or 3 or 4 or 5 or 6 or 7 or 8 or 9 or 10 or 11 or 12 or 13 or 14 or 15 or 16 or 17 or 18 or 19 or 20 or 31 or 32 or 33 or 34 or 35 or 36 or 37 or 38 or 39 or 40 or 41 or 42 or 43 or 44 or 45 or 46 or 47 or 48 or 49 or 50 or 51 or 52 or 53 or 54 or 55 or/and 56 or/and 57 or/and 58 or/and low resource

Annex 2: List of organizations included in search

1. Act Church of Sweden
2. Action Against Hunger
3. Action for Development
4. Adventist Development and Relief Agency International
5. Aga Khan Foundation
6. American Refugee Committee
7. AVSI Foundation
8. Building Foundation for Development Yemen
9. Bureau of Humanitarian Assistance - USA
10. CARE International
11. Center for Reproductive Rights
12. Centers for Disease Control and Prevention
13. Chemonics
14. Columbia University Mailman School of Public Health
15. Columbia University RAISE Initaive, the Heilbrunn Department of Population and Family Health

16. Concern Worldwide
17. CORE group
18. DAI
19. United Kingdom's Foreign, Commonwealth and Development Office
20. Eleanor Crook Foundation
21. ELRHA
22. Emergence and Relief Agency - Arab Medical Union
23. Emergency Nutrition Network
24. Emory University
25. Engender Health
26. European Union Civil Protection and Humanitatian Aid
27. Family Planning 2030
28. FHI360
29. Food and Agriculture Organization
30. Global Alliance for Improved Nutrition
31. Global Justice Center
32. GOAL
33. Guttmacher Institute
34. Helen Keller International
35. HELP Logistics
36. HelpAge International
37. Hope Worldwide
38. IMA World Health
39. IMMAP
40. Institut Bioforce
41. InterAction
42. International Centre for Migration and Health

43. International Committee of the Red Cross
44. International Council of Nurses
45. International Council of Voluntary Agencies
46. International Federation of Red Cross and Red Crescent Societies
47. International Medical Corps
48. International Organisation for Migration
49. International Orthodox Christian Charities
50. International Rescue Committee
51. INTERSOS
52. Ipas
53. JHPIEGO
54. John Hopkins University Center for Humanitarian Health
55. John Snow, Inc
56. Joint Aid Management - JAM International
57. Laerdal Global Health

58. London School of Hygiene & Tropical Medicine
59. Malaysia Medical Relief Society
60. Malteser International
61. Management Sciences for Health
62. Med Global
63. Medair
64. Médecins du Monde
65. Médecins Sans Frontières
66. Medical Teams International
67. Mercy Corps
68. MSI Reproductive Choices

69. Mwada-Gana Foundation
70. Nutrition International
71. Nutrition Works
72. Office of Foreign Disaster Assistance - USA
73. Office of United Nations High Commissioner for Human Rights
74. Oxfam Novib
75. PAI
76. PATH
77. Pathfinder International
78. Plan International
79. Population Council
80. Première Urgence Internationale
81. Public Health Agency of Canada
82. Public Health England
83. Rahnuma-Family Planning Association of Pakistan
84. RedR UK
85. Relief International
86. Reproductive Health Supplies Coalition
87. Research for Health in Humanitarian Crises
88. Samaritans Purse International Disaster Relief
89. Save the Children
90. SNV
91. Sphere
92. Terre des Hommes
93. The Earth Institute - Columbia University
94. The Global Fund to Fight AIDS, Tuberculosis and Malaria
95. The Harvard Humanitarian Initiative

96. UK Med
97. United Nations Children's Fund
98. United Nations High Commissioner for Refugees
99. United Nations Population Fund
100. United Nations System Standing Committee on Nutrition
101. Université Catholique de Louvai
102. University College London
103. United States Agency for International Development
104. Valid International
105. World Food Programme
106. World Health Organization
107. Women Deliver
108. Women's Refugee Commission
109. World Association for Disaster and Emergency Medicine
110. World Vision International
111. Inter-Agency Working Group on Reproductive Health in Crises
112. Global Health Cluster
113. Global Nutrition Cluster
114. Inter-Agency Standing Committee
115. Emergency Nutrition Network
116. United Nations Development Programme

Annex 3: List of included resources

**1**	**A guide to the provision of safe delivery and immediate newborn care in the context of an Ebola outbreak**
**2**	**A safe place to shine**
**3**	**Addressing Child Marriage in Humanitarian Settings: Technical Guide**

4	Addressing Sexual Violence against Men, Boys, and LGBTIQ+ Persons in Humanitarian Settings: A Field-Friendly Guidance Note by Sector
5	Adolescent Sexual and Reproductive Health Toolkit for Humanitarian Settings: 2020 Edition
6	Adolescent sexual and reproductive health in refugee situations: a practical guide to launching interventions in public health programmes
7	Anesthesia Handbook - ICRC
8	Caring for Child Survivors of Sexual Abuse: Guidelines for health and psycho-social providers in humanitarian settings
9	Child Friendly Spaces in Emergencies: A Handbook for Save the Children Staff
10	Clinical care for survivors of Ebola Virus disease interim guidance 2016
11	Clinical Guidelines - Diagnosis and Treatment Manual
12	Clinical Management of Mental, Neurological and Substance Use Conditions in Humanitarian Emergencies
13	Clinical management of patients with viral haemorrhagic fever - a pocket guide for front line health workers
14	Clinical management of rape and intimate partner violence survivors Developing protocols for use in humanitarian settings
15	Communicable diseases and severe food shortage
16	Community Case Management (CCM) in Humanitarian Settings: Guidelines for humanitarian workers
17	Community-based mental health and psychosocial support in humanitarian settings: operational guidelines
18	Consolidated Guideline on Sexual and Reproductive Health and Rights of Women Living with HIV
19	Contraceptive Services in Humanitarian Settings and in the Humanitarian-Development Nexus: Summary of Gaps and Recommendations from a State-of-the-Field Landscaping Assessment
20	Core indicators for adolescent health A regional guide

21	COVID-19 Operational Guidance for Implementation and Adaptation of MHPSS Activities for Children, Adolescents, and Families
22	COVID-19: GBV Risks to Adolescent Girls and Interventions to Protect and Empower Them
23	COVID-19: How to include marginalized and vulnerable people in risk communication and community engagement
24	Current recommendations for treatment of tetanus during humanitarian emergencies A WHO technical note
25	Disabilities among refugees and conflict-affected populations: Report
26	Do's and don'ts in community-based psychosocial programming in regard to sexual violence in conflict-affected settings
27	EARLY CHILDHOOD CARE AND DEVELOPMENT IN EMERGENCIES
28	Early Childhood Development in Emergencies Integrated Programme Guide
29	EARLY CHILDHOOD DEVELOPMENT, NUTRITION AND HEALTH IN EMERGENCIES
30	Ensuring human rights within contraceptive service delivery: Implementation guide
31	Essential Drugs - Practical guide
32	Essential Nutrition Actions: Improving Maternal, Newborn, Infant and Young Child Health and Nutrition - WHO, 2013
33	Essential obstetric and newborn care
34	Evaluation of CFS - Tools and guidance for monitoring and evaluating CFS
35	Evidence and Guidance Note on the Use of Cash and Voucher Assistance for Nutrition Outcomes in Emergencies
36	Fact sheet on the implementation of 2006 WHO Child Growth Standards for emergency nutrition programmes
37	GBV-Mobile-and-Remote-Service-Delivery-Guidelines
38	Gender in Humanitarian Action Handbook
39	Gender-Based Violence in Emergencies: Operational guide

40	GENDER-BASED VIOLENCE PREVENTION AND RESPONSE
41	GLOBAL RAPID GENDER ANALYSIS FOR COVID-19
42	Guidance for Providers Offering Misoprostol-Alone for Abortion Amidst COVID-19
43	Guidance Note Integrating Menstrual Hygiene Management (MHM) into Ebola Response
44	Guidance Note: GBV in Crisis and Post Crisis Settings
45	Guidance on Infant Feeding and HIV in the context of refugees and displaced populations Version 1.1 (2009)
46	Guide to Maternal Newborn and Child Health and Nutrition in Emergencies
47	Guide to Menstrual Hygiene Materials
48	Guidelines for child friendly spaces in emergencies
49	Guidelines for Integrating Gender-Based Violence Interventions in Humanitarian Action: Health
50	Guidelines for Integrating Gender-Based Violence Interventions in Humanitarian Action: Nutrition
51	Guidelines for Selective Feeding: The Management of Malnutrition in Emergencies
52	Guidelines for the Delivery of Antiretroviral Therapy to Migrants and Crisis-Affected Persons in Sub-Saharan Africa
53	Guidelines for the management of pregnant and breastfeeding women in the context of Ebola virus disease
54	Guidelines for the Management of Symptomatic Sexually Transmitted Infections
55	GUIDELINES: FOR THE INTEGRATED MANAGEMENT OF SEVERE ACUTE MALNUTRITION: IN- AND OUT-PATIENT TREATMENT
56	Guidelines: With us and for us, working with young people in humanitarian and protracted crisis
57	Handbook for Coordinating Gender-based violence interventions in humanitarian settings
58	Handbook on pregnancy, childbirth, childhood illnesses, child development and the care of children.

59	HIV AND INFANT FEEDING IN EMERGENCIES: OPERATIONAL GUIDANCE
60	HIV Interventions for Young People in Humanitarian Emergencies
61	HIV self-testing strategic framework: a guide for planning, introducing and scaling up
62	HIV/AIDS Field Guide: A Planning and Practice Guide to Integrating HIV/AIDS into the ICRC's Health Work
63	IASC Guidelines for Addressing HIV in Humanitarian Settings, 2010
64	Identifying & Mitigating Gender-based Violence Risks within the COVID-19 Respons
65	Infant & young child feeding in the context of COVID-19
66	Infant and Young Child Feeding in Emergencies
67	Infant and Young Child Feeding in Emergencies - Guide for Programming
68	Infant and Young Child Feeding in Emergencies (IYCF-E). Programming in The Context Of COVID-19: Considerations for Adaptations
69	Infant and Young Child Feeding in Refugee Situations: A Multi-Sectoral Framework for Action
70	Infant feeding in the context of Ebola – Updated guidance
71	Integrated Management of Child Health: Guide to planning for implementation for IMCI at disrict level
72	Integrating Early Childhood Development (ECD) activities into Nutrition Programmes in Emergencies. Why, What and How
73	Integrating sexual and reproductive health into health emergency and disaster risk management
74	INTER-AGENCY FIELD MANUAL ON REPRODUCTIVE HEALTH IN HUMANITARIAN SETTINGS
75	Interagency Gender-Based Violence Case Management Guidelines: Providing Care and case management Services to Gender-Based Violence Survivors in Humanitarian Settings
76	Interim guideline: nutritional care of children and adults with Ebola virus disease in treatment centers

**77**	Interim Operational Considerations for the feeding support of infants and young children under 2 years of age in Refugee and nigrant transit settings in Europe
**78**	Interim Technical Note PROTECTION FROM SEXUAL EXPLOITATION AND ABUSE (PSEA) DURING COVID-19 RESPONSE
**79**	Malaria control in humanitarian emergencies
**80**	MAMI Care Pathway Package, Version 3 (2021)
**81**	Management of a Cholera Epidemic
**82**	Management of a Measles Epidemic
**83**	Management of Limb Injuries During Disasters and Conflicts
**84**	Managing pertussis outbreaks during humanitarian emergencies
**85**	Manual for the health care of children in humanitarian emergencies
**86**	Manual on Community-based Mental health and psychosocial support in emergencies and displacement
**87**	MENSTRUAL HYGIENE MANAGEMENT IN EMERGENCIES TOOLKIT
**88**	Moderate Acute Malnutrition: A Decision Tool for Emergencies
**89**	Monitoring and Evaluation of Gender-Based Violence Programming in Restricted Environments
**90**	Newborn Health in Humanitarian Settings Field Guide
**91**	Nutrition Cluster Handbook
**92**	Nutrition Humanitarian Needs Analysis Guidance
**93**	Nutrition matters: Guidance for nutrition programming
**94**	Operational considerations for multisectorial mental health and psychological support programmes during the COVID-19 pandemic
**95**	Operational guidance, mental health & psychosocial support programming for refugee operations

96	Operational Guidance: Breastfeeding Counselling In Emergencies
97	PMTCT in Humanitarian Settings
98	Policy Brief Shadows to Spotlight Making Adolescents Visible in Already Collected Data
99	Policy Statement on HIV Testing and Counselling for Refugees and other persons of concern
100	Post-Exposure Prophylaxis (PEP) Treatment: Updated Guidance for Inter-Agency Reproductive Health Kit 3 (2016)
101	PRACTICAL GUIDE FOR A BETTER NUTRITIONAL IMPACT THROUGH INTEGRATED WASH AND NUTRITION PROGRAMS FOR THE ATTENTION OF PRACTITIONERS IN HUMANITARIAN AID AND DEVELOPMENT
102	PREVENTING AND RESPONDING TO CHILD LABOUR IN HUMANITARIAN ACTION
103	Programmatic Guidance for Sexual and Reproductive Health in Humanitarian and Fragile Settings During COVID-19 Pandemic
104	Protection from gender-based violence in food and nutrition security interventions
105	Psychological first aid: Guide for field workers
106	Responding to the health and protection needs of people selling or exchanging sex in humanitarian settings
107	Roadmap to Accelerate Progress for Every Newborn in Humanitarian Settings 2020-2024
108	Stunting in protracted emergency contexts: ENN briefing note
109	Supporting Young Male Refugees and Migrants Who Are Survivors or At Risk of Sexual Violence: A Field Guide for Frontline Workers in Europe
110	Supportive spaces for infant and young child feeding in emergencies. Technical brief
111	Surviving Day One: Caring for Mothers and Newborns in Humanitarian Emergencies on the Day of Childbirth
112	TB
113	The Sphere Handbook

114	Toolkit for Monitoring and Evaluating Adolescent Sexual and Reproductive Health Interventions in Safe Spaces
115	TOOLKIT FOR MONITORING AND EVALUATING GENDER-BASED VIOLENCE INTERVENTIONS ALONG THE RELIEF TO DEVELOPMENT CONTINUUM
116	UNHCR Handbook for the Protection of Women and Girls, First Edition (complete publication)
117	UNICEF Programming Guide Infant and Young Child Feeding
118	Vaccination in humanitarian emergencies implementation guide
119	Very Young Adolescent Sexual & Reproductive Health and Gender Program Design Guide
120	Working with Refugees Engaged in Sex Work: A Guidance Note for Humanitarians
121	Adaptations to the Management of Acute Malnutrition in the Context of COVID-19
122	Sex, age (and more) still matter: Data collection, analysis, and use in humanitarian practice
123	Nutrition of women and adolescent girls in humanitarian contexts: Current state of play
124	Women's nutrition A summary of evidence, policy and practice including adolescent and maternal life stages
125	Sibling Support to Adolescent Girls in Emergencies
126	Solutions Guide for Climate Change, Gender and Health
127	Child Protection and Food Security: An Evidence Review of the Linkages in Humanitarian Settings
128	Ensuring universal access to sexual and reproductive health supplies
129	Sexual and Reproductive Health and Rights during Infectious Disease Outbreaks: Operational Guidance for Humanitarian and Fragile Settings
130	A Multi-Sectoral Framework for Action Roll-out Guide
131	Designing cash and voucher assistance to achieve child protection outcomes in humanitarian settings
132	Improving data for evidence-based decision-making: Reinforcing civil registration and vital statistics and maternal and perinatal death surveillance and response systems interlinkages

**133**	Addressing Gender-Based Violence Across Contexts
**134**	A Comprehensive Approach to Accelerating the Elimination of Female Genital Mutilation
**135**	Considerations for programming School Feeding programmes in Refugee Settings, 2022
**136**	Female Genital Mutilation in Humanitarian Settings in the Arab Region
**137**	Approaching Implementation of Respectful Maternity Care in Humanitarian Settings
**138**	Maternal and Newborn Health During Infectious Disease Outbreaks: Operational Guidance for Humanitarian and Fragile Settings
**139**	Collaborating for Maternal Mental Wellbeing: Technical Brief on Perinatal Mental Health in Humanitarian Settings
**140**	Minimum Service Package MENTAL HEALTH AND PSYCHOSOCIAL SUPPORT
**141**	Analyzing and Improving the Financing of Family Planning Service Delivery in Humanitarian Crises
